# Nanotechnology in Dentin Disinfection: Can We Preserve the Bond?

**DOI:** 10.5005/jp-journals-10005-1590

**Published:** 2019

**Authors:** Prabhakar Attiguppe, Amrita P Tripathi, Suriyan Sugandhan, Saraswathi V Naik, Bikshavathi M Deepak

**Affiliations:** 1 Department of Pedodontics, Bapuji Dental College and Hospital, Davangere, Karnataka, India; 2–5 Department of Pedodontics and Preventive Dentistry, Bapuji Dental College and Hospital, Davangere, Karnataka, India

**Keywords:** Ag–Au (silver–gold) nanoparticles, Chlorhexidine, Microleakage, Resin tag

## Abstract

**Aims:**

The aim of this study is to evaluate the effect of cavity disinfection with 2% chlorhexidine (CHX) and Ag–Au nanoparticles on microleakage and resin tag penetrability of composite restoration under *in vitro* conditions.

**Materials and methods:**

Twenty-five human permanent molars extracted for therapeutic reasons were used in the study. Class V cavity of standard dimension was prepared on the buccal and lingual surfaces of the teeth. The teeth were randomly allocated into two groups based on the cavity disinfectant used: group I being 2% CHX gluconate (chlorhexidina Friedrich and Bianca Mittelstadt (FGM)) and group II being cavity disinfectant containing Ag–Au nanoparticles (nanocare gold). In both the groups, the dentin was etched with 37% phosphoric acid and cavity disinfectants were applied following which dentine bonding agent and composite resin were applied and cured. The specimens were then viewed under a stereomicroscope and a scanning electron microscope for microleakage and resin tag formation, respectively.

**Results:**

The results were statistically analyzed using an independent “*t*” test. No significant difference was seen between the two groups with respect to both microleakage and resin tag penetration values (*p* > 0.05).

**Conclusion:**

The cavity disinfectant containing Ag–Au nanoparticles did not affect the sealing ability and resin tag penetrability of composite resin in permanent molars when compared with 2% CHX.

**How to cite this article:**

Attiguppe P, Tripathi AP, *et al.* Nanotechnology in Dentin Disinfection: Can We Preserve the Bond? Int J Clin Pediatr Dent 2019;12(1):42–46.

## INTRODUCTION

Worldwide, it is estimated that the replacement of an existing restoration accounts for 50–71% of each general dentist's activities.^1^ In most of the clinical situations, replacement of the restorations has been highly associated with the occurrence of secondary caries. It may be caused by residual bacteria left under the restoration, or by the development of a microscopic pathway for leakage past the composite restoration due to polymerization shrinkage which may lead to degradation of bond, increased pulp sensitivity, and pulpal inflammation.^2^ Hence, to reduce the potential for secondary caries development, the use of cavity disinfectant has been gaining a wider acceptance.

Among the various cavity disinfectants, chlorhexidine (CHX) has been widely used because of its antimicrobial property.^3^ It also has an inhibitory effect on the matrix metalloproteinases (MMPs) (against MMPs 2, 8, and 9) in the dentin. This effect can be useful in preventing collagen degradation and disintegration of the bonding interface over time.^4^ Contrary to this, some studies have reported that adhesion could be impaired by a series of previous dentin treatment with CHX.^5,6^ In addition to the above-mentioned adverse effect on adhesion, over the years, the antimicrobial property of CHX has been questioned through various studies.^7,8^

Recently, silver nanoparticles due to its strong antibacterial activity are recommended for cavity disinfection before restoration. Nanoparticles of silver and gold have diversiform size and shape of different surface energies which ensure its antibacterial property against different types of bacteria. Though these materials have good antimicrobial and antifungal activities, there are possibilities that the nanoparticles may agglomerate and create an adverse effect on the bonding of restoration to dentin.^9^

To explore this possibility, this study was conducted to compare and evaluate the effects of two cavity disinfectants, CHX and Ag–Au nanoparticles, on the sealing ability and resin tag formation of composite resins.

## MATERIALS AND METHODS

The study protocol was approved by the Ethics Committee at Institutional Review Board, (under the number 434/2015-16).

### Study Design

Experimental, *in vitro* study, between group study.

### Sample Size Determination^6^

Based on the information available from previous studies, the population means of the experimental and control groups were set at 110 and 90, respectively, with the power of 0.8. The type I error probability was set at 0.05 to obtain a sample size of 15 per group for microleakage. Additional 10 samples were collected for the evaluation of resin tag penetrability.

### Selection of Teeth

Twenty-five human permanent molars extracted for therapeutic reasons were used in the study. Only teeth that were free of caries and restoration and showed no evidence of white spots or cracks were selected.

### Preparation of the Extracted Teeth

The teeth were cleared of debris and stored in a physiological saline solution containing 0.1% thymol at 37°C.

### Evaluation of Microleakage

Class V cavity of standard dimension was prepared on the buccal and lingual surfaces of 15 permanent molars’ tooth using a high-speed airotor handpiece with a straight fissure no. 9 bur and, then, the teeth were hemisectioned mesiodistally with a diamond disk to obtain 30 specimens. The cavities were standardized with William's periodontal probe. Each cavity was approximately 2 mm wide, 1.5 mm deep, and 6 mm long, paralleling the cement–enamel junction. The gingival half of the preparations was extended 0.5 mm below the cementoenamel junction (CEJ). Each preparation was rinsed with distilled water for 20 seconds and dried with a blast of compressed air for 5 seconds. Caution was taken not to over dry the preparation.^10^ The samples were distributed into two experimental groups, consisting of 15 cavities each. All cavities were restored as given below.

In group I, 2% CHX (FGM chlorhexidina), the cavities were etched with 37% phosphoric acid (Scotchbond) for 20 seconds, rinsed for 20 seconds with air–water syringe, and dried with compressed air for 10 seconds, this was followed by the application of 2% CHX disinfectant on the cavity walls and floor using disposable microapplicator tips for 60 seconds.^11^ Then, prime and bond NT was applied for 20 seconds, lightly air dried for 5 seconds, and light cured for 10 seconds using the LED curing unit (BLUEDENT LED smart) with a light output of 1,300 mW/cm^2^ at a pulsed mode. All the cavities were filled in 2 mm increments of composite restorative (FILTEK 350 XT, 3M ESPE). Each increment was cured for 20 seconds by the previous light cure unit. The composite restorations were finished 24 hours after the completion of restorations using diamond finishing bur (Diatech Coltene/WhaledentAG, Switzerland) under water and were polished with Sof-lex Disks.

In group II, cavities were prepared in a similar manner as described previously. After the preparation of the class V cavity, etching was carried out with 37% phosphoric acid; as described in group I, nanocare gold (Dental Nanotechnology, SA) was applied using a disposable applicator tip in the amount of five drops and left undisturbed to evaporate for approximately 3 minutes. Following this, the prime and bond bonding NT was applied and cavities were restored with a composite restoration as described previously. All teeth were stored at 37°C for 24 hours in distilled water. The teeth were thermocycled in water between 5°C and 55°C with a dwell time of 30 seconds for a total of 500 cycles. After thermocycling, the root apices were sealed with the modeling wax and the teeth completely sealed to within 1 mm margin of the restoration with two coats of fingernail polish. The specimens were immersed in methylene blue dye at 37°C for 24 hours. After staining, the teeth were rinsed off to remove the residual dye. The teeth were sectioned buccolingually in the approximate center of the restorations with a diamond disk in a straight air motor handpiece. Microleakage was assessed for both occlusal and gingival margins by an examiner blinded to the test using a stereomicroscope (Leica Wild M3Z, Germany) at ×30 magnification and subjected to statistical analysis.

The scoring system was used similar to that used by Meiers and Kresin (1996):^3^

Microleakage scores:

- 0 = no leakage.
- 1 = penetration less than ½ the length of the occlusal/gingival wall.
- 2 = penetration greater than ½ the length of the occlusal/gingival wall.
- 3 = penetration up to and along the axial wall.

### Evaluation of Resin Tag Formation

**Fig. 1:**
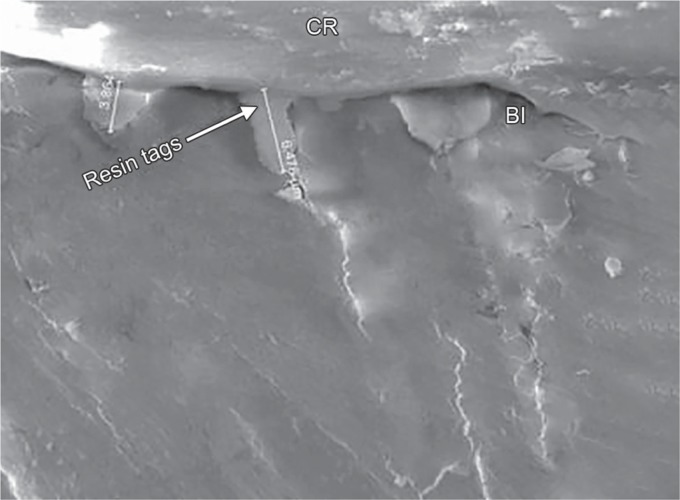
Representative sample from Group II after FEI Scanning electron microscope evaluation showing resin tags in μm at 5000× mag

Ten extracted human permanent molars were selected. Class V cavities were prepared on the buccal of all the teeth in a similar manner as performed for the evaluation of microleakage. Each preparation was rinsed with distilled water for 20 seconds and dried for 20 seconds. The cavities were etched with 37% phosphoric acid (Scotchbond) for 20 seconds rinsed for 20 seconds with an air–water syringe and dried with compressed air for 10 seconds, this was followed by the application of 2% CHX disinfectant (2% chlorhexidina, FGM) on the cavity walls and floor for group I (*n* = 5) and Ag–Au nanoparticles (nanocare gold) for group II (*n* = 5) using disposable microapplicator tips, in a similar method carried out for microleakage. Following this, prime and bond NT (Dentsply) bonding agent was applied to the preparations according to the instruction from the manufacturer and restored with composite resin (FILTEK Z 350 XT, 3M ESPE). The specimens were sectioned buccolingually in the approximate center of the restorations with a low-speed diamond with continuous spraying of water. After the sectioning of the teeth, specimens were polished using the silicon carbide paper of size 320 and 600 grit. Sections were fixed by immersing them in 10% formalin for 12 hours and, then, the sections were viewed under a scanning electron microscope (FEI Quanta 200) and the measurement of resin tag length was evaluated in micron meters (Fig. 1) and the results were subjected to statistical analysis.

## RESULTS

**Fig. 2:**
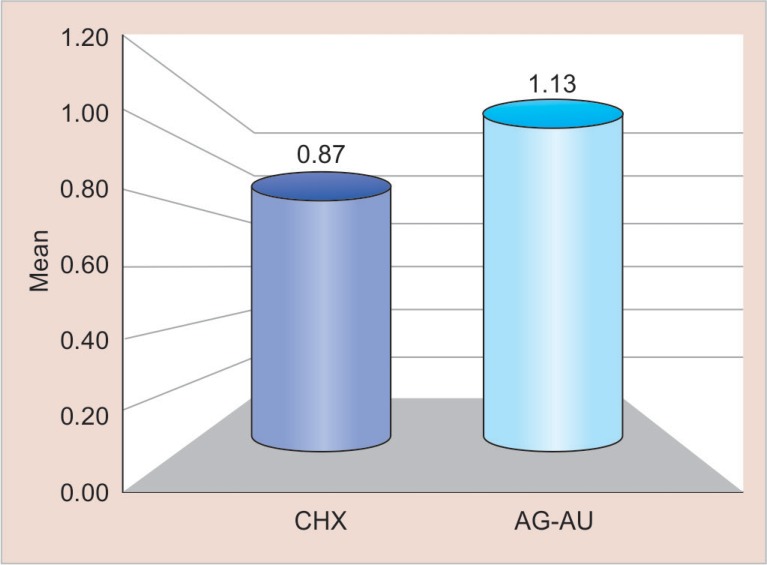
Mean comparison of microleakage observed using cavity disinfectants containing CHX and Ag–Au nanoparticles at gingival margin

**Fig. 3:**
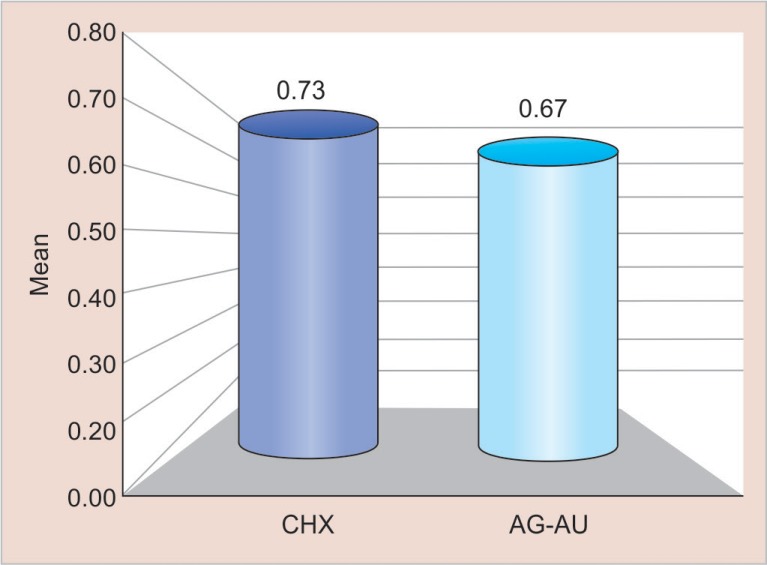
Mean comparison of microleakage observed using cavity disinfectants containing CHX and Ag–Au nanoparticles at occlusal margin

All the analyses were carried out with SPSS 20.0 software. Means and standard deviations values of both the groups are shown in Tables 1 to 3. The scores for microleakage along the occlusal and gingival margins in both the groups were compared using an independent “*t*” test. The level of significance was established as *p* < 0.05, for all the tests. Pair wise comparisons for microleakage among the two experimental groups showed no statistically significant differences as shown in Figures 2 and 3 with a *p* value of 0.363 and 0.702 at gingival and occlusal margins, respectively.

**Fig. 4:**
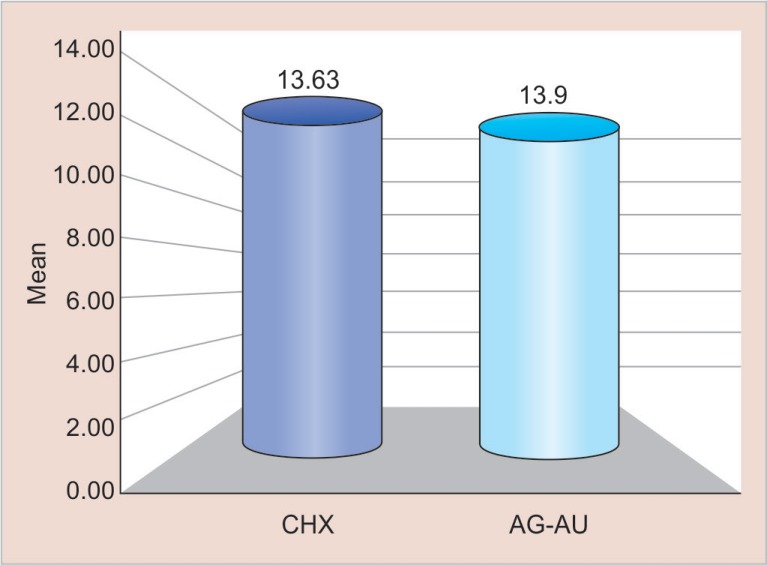
Mean comparison of resin tags observed using cavity disinfectants containing CHX and Ag–Au nanoparticles

For the resin tag formation, the “*p*” value of 0.859 was obtained which was indicative that there was no statistically significant difference in the mean penetrability of both the groups. Table 3 and Figure 4 elucidate the occurrence of penetrability (resin tag lengths) in both groups. The group I showed the mean value of penetrability of 13.63 μm, whereas the group II showed the mean value of penetrability of 13.09 μm.

**Table 1:** Mean comparison of microleakage observed using cavity disinfectants containing CHX and Ag–Au nanoparticles at gingival margin

*Variables*	*Groups*	*Min*	*Max*	*Mean*	SD*	*Difference*	*I value*	*p value*
						*Mean ± SD*		
Gingival	CHX	0.00	2.00	0.87	0.74	0.26 ± 0.09	0.925	0.363
	Ag–Au	0.00	3.00	1.13	0.83			NS

**Table 2:** Mean comparison of microleakage observed using cavity disinfectants containing CHX and Ag–Au nanoparticles at occlusal margin

*Variables*	*Groups*	*Min*	*Max*	*Mean*	SD*	*Difference*	*I value*	*p value*
						*Mean ± SD*		
Occlusal	CHX	0.00	1.00	0.73	0.46	0.6 ± 0.03	0.386	0.702
	Ag–Au	0.00	1.00	0.67	0.49			NS

**Table 3:** Mean comparison of resin tag formation observed using cavity disinfectants containing CHX and Ag–Au nanoparticles

*Variables*	*Groups*	*Min*	*Max*	*Mean*	SD*	*Difference*	*I value*	*p value*
						*Mean ± SD*		
Resin tag formation (micro-meter)	CHX	9.69	18.5	13.63	3.88	0.54 ± 1.45	0.183	0.859
	Ag–Au	7.01	18.9	13.09	5.33			NS

## DISCUSSION

In the present study, following cavity disinfection with 2% CHX digluconate (chlorhexidina, FGM) and Ag–Au nanoparticles (nanocare gold), two important parameters, microleakage and resin tag penetrability, were evaluated. CHX which is a gold standard was selected as a control because it is one of the most widely used antimicrobial agents in oral health.

**Fig. 5:**
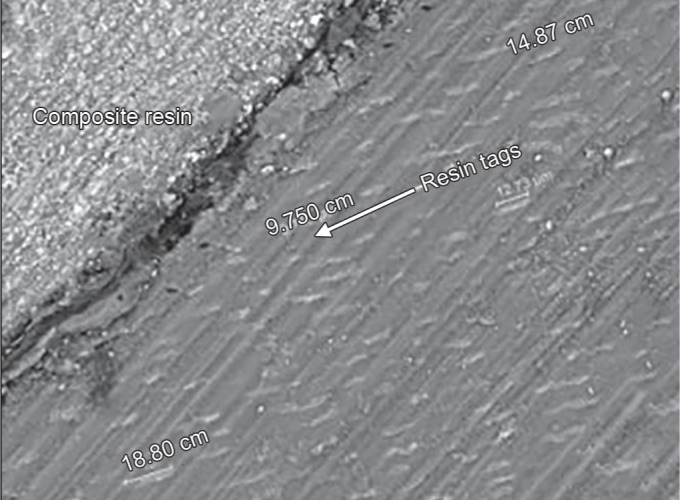
Representative sample from Group I after FEI scanning electron microscope evaluation showing length of the resin tag ranging from 9.75 μm to 18.90 μm (1000× mag)

**Fig. 6:**
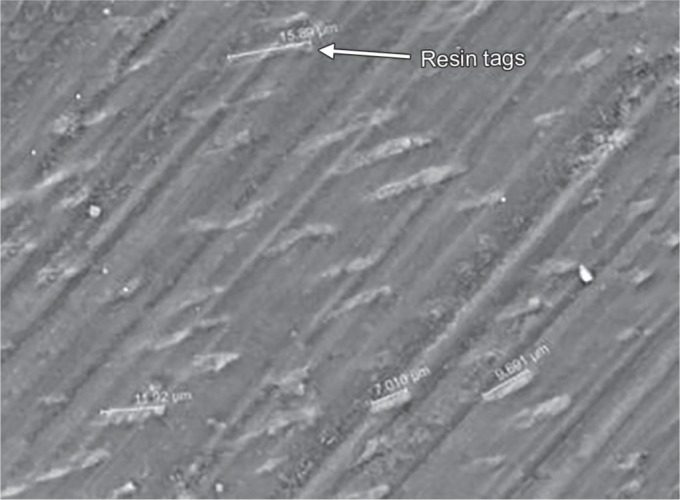
Representative sample from Group II after FEI Scanning electron microscope evaluation showing length of the resin tag ranging from 7.010 μm to 15.89 μm (2000× mag)

The results of the present study indicated that there was no statistically significant difference observed (*p* = 0.702) with respect to the scores of microleakage between the two groups at the occlusal and gingival margin. When the enamel and gingival margin in the two experimental groups were compared, microleakage was higher in the gingival margin compared with the enamel margin. These results were consistent with other studies where there was more microleakage at the gingival margin. The rationale for this finding is due to thinner enamel margin at the gingival third than the occlusal third and the thickness of the incisal enamel prevented permeability resulting in a more resistant surface to dye penetration.^3,12,13^ With respect to the resin tag penetrability, it was found that that there was no statistically significant difference (*p* = 0.859) between the lengths of the resin tags formation between the two groups. The length of resin tags formation in group I varied from 9.750 to 18.90 μm, while in group II, it ranged from 7.01 to 15.89 μm (Figs 5 and 6).

In the present study, nanocare gold cavity disinfectant did not affect the sealing ability and resin tag formation of the composite to dentin. The results were in accordance with the study conducted by Porenczuk et al., where nanocare gold did not influence the adhesive's bond strength to dentin.^14^ The reason for this could be, nanocare gold cavity disinfectant is composed of the numerous spherical nanoparticles (round, discoid) which were of the mean size of 48 nm. Lohbauer et al. suggested that the spherical shape of the nanoparticles provides with only one point of contact, decreasing the tendency of agglomeration. In addition, the manufacturer claims that the metal nanoparticles are dispersed in a liquid carrier such as isopropanol.^15^ This provides an added advantage as nanoparticles (NPs) agglomeration can be prevented by their dissolution in a liquid carrier like methanol and isopropanol. Also, different sizes and shapes of nanoparticles may act as inorganic fillers similar to hybrid composites, this may be the feature which enables nanocare gold to preserve the restorative material's physical properties.^9^

In the present study, the etch and rinse adhesive system was used and CHX and Ag–Au nanoparticles (nanocare gold) were applied after acid etching in the solution form since various authors have reported about the benefits that were gained after the application of CHX on the acid-etched dentin. Certain CHX properties, including strong positive ionic charge; ready binding to phosphate groups; strong affinity to the tooth surface, which is increased by acid etching; and, finally, an increase in surface-free energy of enamel and perhaps dentin, are the likely reasons responsible for the good resin–dentin bond strengths obtained when CHX is applied after acid etching.^16,17^

The adhesive material used in this study, the prime and bond NT, uses acetone as a solvent that removes the residual moisture, enhances resin wetting, and may counteract the adverse effects of organic contamination on bonding to the tooth structure.

Microleakage was assessed because it is an important consideration in assessing the adhesion of materials to both enamel and dentin. As microleakage may be influenced by factors such as thermal changes; in the present study, after composite restoration, the specimens were subjected to thermocycling because it is a widely used method to simulate temperature changes that take place in the oral environment. For the present study, 500 thermal cycles between 5°C and 55°C were used. Radovic et al. also used 500 cycles, as it was based on the current ISO standard.^18^ The evaluation of resin tag determines the formation of an acid resistant, resin impregnated hybrid layer which seems to depend on the penetrating ability of resin into the etched dentin surface and also on conditioning and permeability of dentinal surface.

Reviewing the literature and correlating the inferences with the results of this study shows us that the application of nanocare gold for cavity disinfection does not interfere with the bonding, sealing ability, and resin tag penetration of the composite restorations. Further, *in vivo* studies need to be conducted to examine the interaction and long-term effect of nanocare gold with the other two step and self-etch adhesive system.

## CONCLUSION

Within the parameters tested in the present intervention, the use of cavity disinfectant containing silver and gold nanoparticles (nanocare gold) with the etch and rinse system can be recommended prior to placement of composite restoration as it does not have any adverse effect on the sealing ability and resin tag penetration of resin restorations.

## CLINICAL SIGNIFICANCE

The use of cavity disinfectant containing Ag–Au nanoparticles with the etch and rinse system can be preferred as it satisfies the ideal property of a cavity disinfectant which includes excellent antimicrobial action and non-detrimental effect on the sealing ability and resin tag penetrability.
